# Compositional studies and Biological activities of *Perovskia abrotanoides* Kar. oils

**DOI:** 10.1186/0717-6287-47-12

**Published:** 2014-04-01

**Authors:** Sadaf Naz Ashraf, Muhammad Zubair, Komal Rizwan, Rasool Bakhsh Tareen, Nasir Rasool, Muhammad Zia-Ul-Haq, Sezai Ercisli

**Affiliations:** Department of Chemistry, Government College University, Faisalabad, 38000 Pakistan; Department of Botany, University of Balochistan, Quetta, Pakistan; The Patent Office, Karachi, Pakistan; Ataturk University Agricultural Facultu Department of Horticulture, 25240 Erzurum, Turkey

**Keywords:** *Perovskia abrotanoides*, Essential oil, Fixed oil, Antioxidant capacity, Antimicrobial activity

## Abstract

**Background:**

Current study has been designed to evaluate the chemical composition of essential and fixed oils from stem and leaves of *Perovskia abrotanoides* and antioxidant and antimicrobial activities of these oils.

**Results:**

GC-MS analysis of essential oil identified 19 compounds with (E)-9-dodecenal being the major component in stem and hexadecanoic acid in leaves. In contrast, GC-MS analysis of fixed oil showed 40 constituents with α-amyrin the major component in stem and α-copaene in leaves. The antioxidant activity showed the highest value of 76.7% in essential oil from leaves in comparison with fixed oil from stem (45.9%) through inhibition of peroxidation in linoleic acid system. The antimicrobial assay tested on different microorganisms (e.g. *E. coli, S. aureus, B. cereus, Nitrospira, S. epidermis, A. niger, A. flavus and C. albicans*) showed the higher inhibition zone at essential oil from leaves (15.2 mm on *B. cereus*) as compared to fixed oil from stem (8.34 mm on *S. aureus*) and leaves (11.2 mm on *S. aureus*).

**Conclusions:**

The present study revealed the fact that essential oil analyzed from Perovskia abrotanoides stem and leaves could be a promising source of natural products with potential antioxidant and antimicrobial activities, as compared to fixed oil.

## Introduction

*Perovskia abrotanoides* (Lamiaceae) locally known as *hoosh, visk, brazambal*, *domou*, and *gevereh*[1–4] is a medicinally important plant found in Baluchistan province and northern areas of Pakistan. The plant is used by local communities for treatment of typhoid, headache, gonorrhea, vomiting, motion, toothache, atherosclerosis, cardiovascular diseases, liver fibrosis, and cough [5–7]. It has sedative, analgesic, antiseptic and cooling effect [5, 8, 9]. Herbal tea of this plant is used in curing infection problems and painful urination [10].

Some of the pharmacological effects of plant such as antiplasmodial, antiinflammatory and cytotoxic effects have also been reported [11–13]. Its antioxidant performance including heart enhancing and optimized performance as cell toxicity in pathogens, viruses and cancer cells were also reported [5, 14]. Essential and fixed oils of *Perovskia abrotanoides* plays an important role in protection of stored grains and showed to be effective in washing wounds, anti-ring worm, dermal parasites, anti-fungus and anti-hypoxia [14–17]. Despite its multipurpose usage, little data exists on chemical composition as well as biological activities of this plant. Therefore this study was designed to investigate composition of essential and fixed oil and antioxidant and antimicrobial activities of stem and leaves of *Perovskia abrotanoides.*

## Results and discussion

The diversity of agrogeoclimatic conditions of Pakistan offers the broadest array of flora. This rich floral biodiversity of Pakistan constitutes an impressive pool for ‘natural food and healing’ from which the indigenous communities select ingredients for food and prepare herbal recipes for the treatment, management and control of their various ailments. It is believed that antimicrobials and antioxidants of plant origin are without side effects as compared to synthetic drugs and have an enormous therapeutic potential to heal many infections and diseases. Much of the potential of these botanicals is however still unearthed. In current research, a less explored medicinal plant found in Pakistan has been investigated for compositional studies and biological activities.

### GC-MS analysis of essential and fixed oils

**Figure 1:**
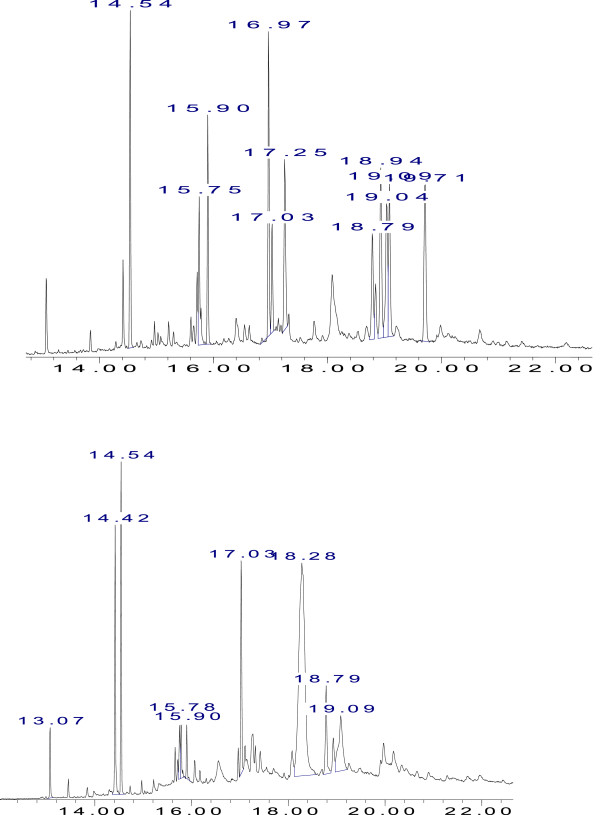
**GC-MS product distribution of the** ***P. abrotanoides*** **essential oil from stem (top) compared with essential oil from leaves (bottom).**

Qualitative and quantitative GC-MS analysis of the essential and fixed oils were performed in order to identify different compounds in the oils. The GC-MS analysis identified 13 and 15 compounds in the essential oils of *P. abrotanoides* stem and leaves respectively. Essential oils of plant (stem, leaves) consisted of a mixture of different classes of compounds. The major components found in essential oil of stem were (E)-9-dodecenal (66.5%), octadecanoic acid, methyl ester (8.37%), 2,2,5,5-tetramethylhexane (3.96%), while in leaves: hexadecanoic acid, methyl ester (27.79%), lupeol (21.5%), octadecenoic acid, methyl ester (18.45%), eicosane (6.22%) and tetradecane (5.19%) were present in higher concentrations. Considerable amount of some other constituents was also present in the plant essential oils. (Table 1, Figure 1). The major component found in essential oil was (E)-9-dodecenal followed by 5,6-dimethylheptadecane, octadecanoic acid and tetradecane in stem and hexadecanoic acid followed by 5-β-cholestan-3α-ol, and hexatricontane in leaves (around 51% of the total compounds). The fixed oils of stem and leaves were analyzed by GC-MS to monitor their compositions (Table 1). A total of fifty constituents were identified in the fixed oils of *P. abrotanoides* which represented the 86% (stem) and 86.35% (leaves) composition of the total oil. In stem oil the major constituents (> 5%) were α-amyrin (47.01%), α-amyrenone (11.8%) and isopropyl-hexadecanoate (6.56%) while in leaves α-copaene (10.99%), trans-phytol (7.33%), isopropyl-hexadecanoate (6.67%), unidentified (5.63%) and α-amyrenone (5.19%) were present in major concentration. The composition of essential and fixed oils content showed variations in different parts (stem and leaves) of the plant. There are some reports in the literature on the chemical composition of the different chemotypes of *P. abrotanoides* essential oil from different countries. Camphor, α-pinene, o-cimene, 1,8-cineol, camphene, borneol, β-pinene, α-humulene, caryophyllene and some other components have already been reported from aerial parts of *P. abrotanoides*. Camphor, borneol, α-terpineol, bornyl acetate, α-humulene and α-cadinol were present in high concentration in the oil of stem, leaves, flowers and roots of *P. abrotanoides*[18–22]. Our results are in partial agreement with the earlier reports of this plant. It is well known that the essential and fixed oil contents and composition depend on several factors such as different genotype, agronomic practices employed, climatological factors, development stages of plant parts analysed, growing condition, season, post-harvest storage and processing conditions and solvent used for extraction*.* These factors may explain the differences found among our samples and those analyzed in previous studies. Thereof we have identified some of the similar compounds which reported before in oils of this plant and some new compounds have also reported in our study which are not reported earlier.

**Table 1:** **GC-MS product distribution and relative proportion (wt% of total compounds) of dry weight essential oil (stem and leaves) from** ***Perovskia abrotanoides*** **(in mg/g oil)**

RI	Chemical constituents	% Composition			
		Essential oil		Fixed oil	
		Stem	Leaves	Stem	Leaves
820	2,2,3,4-tetramethylpentane	0.46	--	--	--
850	t*	--	0.77	--	--
922	2,4,6-trimethyloctane	0.76	--	--	--
973	Nonane, 5-(2-methylpropyl)	--	1.07	--	--
1000	t*	--	--	0.33	--
1033	Eucalyptol	--	--	--	1.26
1052	2,2,5,5- tetramethylhexane	3.96	--	--	--
1125	Ethylbenzene	--	--	--	3.08
1143	Camphor	--	--	--	1.54
1165	Borneol	--	--	--	2.12
1175	S-propyl methanesulfonothioate	3.0	--		
1189	α terpineol	--	--	--	1.07
1200	1-bromo dodecane	--	--	0.11	--
1236	2-pentylfuran	--	--	0.52	3.05
1291	Bornyl acetate	--	--	--	0.7
1352	Terpinyl acetate	--	--	--	1.26
1362	Geranyl acetate	--	--	--	0.83
1399	Tetradecane	2.41	5.19	--	--
1403	(E)-9-dodecenal	66.5	--	--	--
1404	δ-caryophyllene	--	--	--	0.7
1416	α-copaene	--	--	--	10.99
1424	Caryophyllene	t*	--	--	0.95
1454	1,4,7-cycloundecatriene,1,5,9,9-tetramethyl, Z,Z,Z-	--	--	--	0.82
1500	Pentadecane	--	3.97	t*	--
1532	Epiglobulol	--	--	0.65	4.95
1642	Cubenol	--	--	--	1.64
1654	α-cadinol	--	--	0.48	--
1659	β-eudesmol	--	--	0.42	--
1719	8-hexylpentadecane	--	--	--	1.16
1720	Tetradecanoic acid	--	--	0.17	--
1729	4-(E)-3-hydoxyprop-1-enyl)-2-methoxyphenol	--	--	1.26	1.93
1750	t*	--	--	0.33	--
1800	t*	--	--	--	0.81
1800	Cis-9-hexadecenal	--	--	2.23	--
1822	5,6-dimethylheptadecane	2.94	--	--	--
1834	Unidentified	--	--	2.61	2.06
1847	2-pentadecanone, 6,10,14-trimethyl	--	--	--	0.79
1854	5-methyloctadecane	--	--	0.48	3.21
1914	Anthranilic acid	--	--	--	1.04
1949	Heptadec-1-ol	--	--	--	0.99
1962	Hexadecanoic acid, methyl ester	--	27.79	--	--
2000	Eicosane	--	6.22	--	--
2000	Unidentified	--	--	--	5.63
2010	Unidentified	--	--	--	1.28
2012	Isopropyl hexadecanoate	--	--	6.56	6.67
2080	Octadecanoic acid, 17- methyl ester	--	2.19	--	--
2088	Octadecanoic acid, methyl ester	8.37	18.45	--	--
2100	Unidentified	1.93	--	--	--
2110	t*	--	--	--	0.69
2111	Trans phytol	--	--	2.03	7.33
2138	7-methoxy-8-(3-methyl-2-butenyl)-2H-chromen-2-one	--	--	2.29	--
2161	Oleic acid	--	--	--	3.31
2200	Stearic acid	--	--	0.33	--
2221	Nonadecanoic acid	--	--	0.6	--
2240	Androst-2-en-17-one	--	--	2.18	---
2330	Trans-ferruginol	--	--	0.48	3.06
2400	Tetracosane	--	--	0.17	--
2430	3-hydroxyandrostan-17-one	--	--	--	2.06
2435	t*	--	--	--	0.88
2526	Docosanoic acid	--	2.47	--	--
2700	Unidentified	--	1.1	--	--
2730	Tetracosanoic acid	--	2.62	--	--
3011	t*	0.53	--	--	
3098	5.β-cholestan-3α-ol	--	1.46	--	--
3400	Tetratriacontane	--	--	0.14	--
3408	β sitosterol	--	--	2.82	--
3654	1-(+)-Ascorbic acid 2,6-dihexadecanoate	3.0	--	--	--
3654	Unidentified	--	--	--	3.3
3600	Hexatriacontane	--	0.69	--	--
4000	Unidentified	2.51	1.99	--	--
4050	α-amyrenone	--	--	11.8	5.19
4160	α-amyrin	--	--	47.01	--
4175	Lupeol	--	21.5	--	--

t* = traces.

Compounds identification was carried on the basis of Retention indices and EI/MS.

### Antioxidant activity

Antioxidants are an important part of the defense system of the human body and help to cope with oxidative stress caused by reactive oxygen species. Plants are important sources of antioxidants and there is increasing interest in antioxidant analysis of plants [23]. DPPH• is increasingly used for quickly assessing the ability of antioxidants to transfer the labile H atoms to radicals [24]. This hydrogen donation ability leads towards formation of stable complex of free radicals, resulting in termination of damages caused by these radicals. The essential and fixed oils were screened for their possible antioxidant activity by DPPH radical scavenging (IC_50_) (Table 2). The stem essential oil showed potential DPPH radical scavenging activity (IC_50_ = 17.9 μg/mL) then leaves essential oil (IC_50_ = 45.4 μg/mL) and the fixed oil of leaves exhibited high DPPH radical scavenging activity (IC_50_ = 62.5 μg/mL) than fixed oil of stem (IC_50_ = 73.1 μg/mL). Standard Antioxidant compound BHT showed highest DPPH radical scavenging activity (IC_50_ = 8.78 μg/mL). Antioxidant effect of a plant extract and its fixed or essential oil is mainly due to various bioactive compounds like flavonoids, phenolic acids, tannins and diterpenes.

**Table 2:** **% Yield and Antioxidant activity of** ***Perovskia abrotanoides*** essential and fixed oils*

Plant parts and tested samples		% yield (g/100 g)	DPPH radical scavenging (IC _50_ ) (μg/mL)	Inhibition of peroxidataion in linoleic acid system (%)
Stem	Essential oil	2.13 ± 0.06	17.9 ± 0.004	61.1 ± 0.87
	Fixed oil	2.96 ± 0.04	73.1 ± 0.007	45.9 ± 1.74
Leaves	Essential oil	2.86 ± 0.05	45.4 ± 0.01	76.7 ± 0.87
	Fixed oil	3.61 ± 0.10	62.5 ± 0.05	47.4 ± 0.87
Standard	BHT	--	8.78 ± 0.10	90.4 ± 0.87

*Values are mean ± S.D. of three separate experiments (P < 0.05).

The percent inhibition of linoleic acid peroxidation was observed for plant essential and fixed oils whereas synthetic BHT provided inhibition at the level of 90.4%. Essential oil of leaves exhibited highest % inhibition of peroxidation (76.4%) followed by stem essential oil (61.1%), leaves fixed oil (47.4%) and stem fixed oil (45.9%). When the results of DPPH scavenging activity and the percent inhibition of linoleic acid oxidation were compared with standard BHT, all the oils showed significantly (*p* < 0.05) minor activity. Essential oils have the efficacy to reduce the peroxide formation during incubation in linoleic acid system as per our previous studies [25–27].

**Figure 2:**
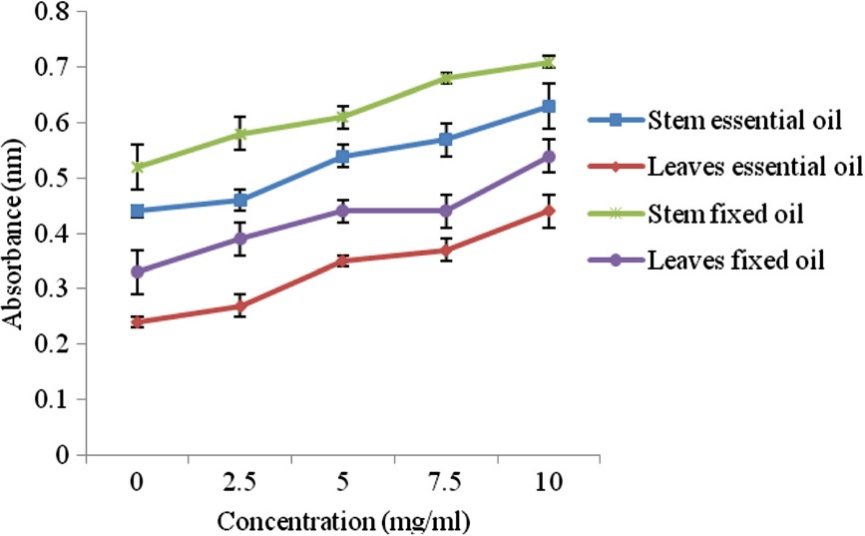
**Reducing potential of essential and fixed oils of** ***P. abrotanoides*** **(stem and leaves).**

The reducing potential of essential and fixed oils of *P. abrotanoides* was also investigated at various concentrations (2.5-10 mg/mL) and absorbance recorded at 700 nm (Figure 2). The order of reducing potential of *P. abrotanoides* was found as: leaves essential oil > leaves fixed oil > stem essential oil > stem fixed oil. Reducing power of different plants and essential oils has already been reported in literature. Plants have reducing power due to the presence of phenolic compounds [28–31]. The results showed that antioxidant activities of essential oil was much higher in respect with fixed oil, which could be due to the higher concentration of (E)-9-dodecenel from stem and hexadecanoic acid from leaves determined by GC-MS analysis.

### Antimicrobial activity

The antimicrobial activity of the *P. abrotanoides* oils was assessed (Table 3). The results from the disc diffusion method, followed by measurement of minimum inhibitory concentration (MIC), indicated that the Stem essential oil showed good inhibitory activity against *Nitrospira sp.* and *A. flavus* (IZ = 9.76, 9.94 mm; MIC = 19.6, 14.5 mg/mL) and it was inactive against *E. coli, S. aureus, B. cereus, S. epidermidis* and *C. albicans*. Stem fixed oil showed potent activity against *C. albicans* (IZ = 25.2 mm; MIC = 1.26 mg/mL) and moderate activity against *E. coli, S. aureus* (IZ = 12.9, 8.34 mm; MIC = 7.95, 14.2 mg/mL). Stem fixed oil exhibited no inhibitory activity against *B. cereus, Nitrospira sp., S. epidermidis, A. niger, A. flavus*. Leaves essential oil was inactive against *E.coli, S. aureus*, *Nitrospira, S. epidermidis, A. flavus* and it moderately inhibited the growth of *B. cerus. A. niger, C. albicans*. Leaves fixed oil was inactive against *E. coli, B. cerus, Nitrospira.* And it showed potent activity against *C. albicans* (IZ = 24.2 mm, MIC = 1.93 mg/mL) and moderately inhibited the growth of other microbes. For the comparison of results Novidate and Fungone were used as positive control for bacterial and fungal strains respectively. The standard drugs showed higher activity on the microbes than the plant oils (Table 3). The standard antibiotics were highly purified chemical compounds so there activity was more as compared to the oils of leaves and stem. For the comparison of results, Novidate and Fungone were used as positive control for bacterial and fungal strains respectively. The standard drugs showed higher activity on the microbes than the plant oils. Our previous studies have found that essential oil obtained from different parts (flowers, leaves, stem, roots) of *P. abrotanoides* possessed potential antimicrobial activity against *S. aureus, B. cereus, S. typhi* and *C. albicans* and a decreased activity against *A. niger*[25–27], which could be a therapeutically approach for acute kidney injury.

**Table 3:** **Antimicrobial activity of** ***Perovskia abrotanoides*** essential and fixed oils*

Diameter of Inhibition Zone (IZ, mm)						
Tested microrganisms	Stem		Leaves		Standard drugs	
	Essential oil	Fixed oil	Essential oil	Fixed oil	Novidate	Fungone
*E. coli*	-	12.9 ± 0.03	-	-	27.7 ± 0.02	-
*S. aureus*	-	8.34 ± 0.03	-	11.2 ± 0.04	21.2 ± 0.03	-
*B. cereus*	-	-	15.2 ± 0.05	-	24.5 ± 0.02	-
*Nitrospira sp.*	9.76 ± 0.01	-	-	-	31.7 ± 0.04	-
*S. epidermidis*	-	-	-	14.2 ± 0.07	23.4 ± 0.01	-
*A. niger*	-	-	10.6 ± 0.005	8.46 ± 0.01	-	30.2 ± 0.06
*A. flavus*	9.94 ± 0.03	-	-	9.64 ± 0.02	-	28.4 ± 0.01
*C. albicans*	-	8.07 ± 0.02	9.78 ± 0.01	8.32 ± 0.03	-	30.2 ± 0.01
Minimum Inhibitory Concentration (MIC, mg/mL)						
*E. coli*	-	7.95 ± 0.02	-	-	0.77 ± 0.01	-
*S. aureus*	-	14.2 ± 0.03	-	9.40 ± 0.05	1.06 ± 0.02	-
*B. cereus*	-	-	5.75 ± 0.04	-	0.95 ± 0.03	-
*Nitrospira sp.*	19.6 ± 0.05	-	-	-	0.28 ± 0.01	-
*S. epidermidis*	-	-	-	5.25 ± 0.02	0.88 ± 0.03	-
*A. niger*	-	-	11.4 ± 0.02	18.4 ± 0.01	-	0.47 ± 0.02
*A. flavus*	14.5 ± 0.01	-	-	17.1 ± 0.03	-	0.24 ± 0.01
*C. albicans*	-	1.26 ± 0.01	11.7 ± 0.08	1.93 ± 0.02	-	0.17 ± 0.02

*Values are mean ± S.D of three separate experiments (P < 0.05).

## Conclusions

We have investigated two different types of *Perovskia abrotanoides* oils. The presence of different compounds founded by GC-MS analysis, rendered the essential oil very efficient in antioxidant and antimicrobial capacity in respect with fixed oil which could be due to the presence of unsaturated fatty acids and anthocyanins compounds. We believe that the compounds, in particular (E)-9-dodecenal and hexadecanoic acid from essential oil are directly involved in antioxidant and antimicrobial processes. Finally, our study revealed the fact that essential oil analysed from *Perovskia abrotanoides* stem and leaves could be a promising source of natural products with potential antioxidant and antimicrobial activities in respect with fixed oil.

## Methods

### Collection of plant material

The *Perovskia abrotanoides* whole plant was collected and deposited from Quetta and Ziarat Valley and further identified by Dr. Rasool Bukhsh Tareen, Department of Botany, University of Baluchistan, Quetta, Pakistan.

### Essential oil extraction

Dried powdered of plant material (100 g) was subjected to hydro-distillation for 5 h using a Clevenger type apparatus for extraction of stem and leaves from essential oils. The extracted essential oil was dried over anhydrous Na_2_SO_4_, filtered and stored in a vial at 4°C until further analysis.

### Fixed Oil extraction

The fixed oil was extracted following the method of Ajayi and contributors [32]. Briefly, 100 g of the shade dried powdered stem and leaves of plant were separately extracted in 250 mL *n-*hexane (99.9% purity) solvent by using a Soxhlet extractor for 6 h and then the extra-solvent was removed by distillation under reduced pressure in a rotary evaporator at 35°C and the pure oil was kept at 4°C in the dark.

### GC-MS analysis of essential and fixed oils

Essential and fixed oils were analysed by GC-MS (QP2010, SHIMADZU, Japan) using an Agilent GC 6890 N model (1 μL sample injected, split 1:50 column flow 1.0 mL/min., program temp. 200°C, rate 10°C/min) coupled with a quadrupolar MS 5973. GC was equipped with capillary column (30 m × 0.25 mm; film thickness 0.25 μm). Oven temperature was kept at 45°C first for 5 min, and then raised at 325°C at a 15°C/min for another 5 min. Helium gas was then employed at a flow rate of 1.1 mL/min (60 KPa pressure; 38.2 cm/sec linear velocity). The identification of components was based on comparison of their mass spectra with those of NIST mass spectral library [33].

### Antioxidant activity

#### The 2-diphenyl-1-picrylhydrazyl (DPPH) radical scavenging assay

The antioxidant activity of the oils was assessed by their ability to scavenging DPPH stable radicals as reported earlier [34]. The samples (10 to 500 μg/mL) were mixed with DPPH solution (1 mL; 90 μM) and then with methanol (95%) to a final volume of 4 mL. Synthetic antioxidant, butylated hydroxytoluene (BHT) was used as control. After 1 h incubation period at room temperature, the absorbance was recorded at 515 nm. Percent radical scavenging concentration was calculated using the following formula:


Where:

A_blank_ = absorbance of the control

A_sample_ = absorbance of the test samples

#### Inhibition of linoleic acid peroxidation

The antioxidant activity of *Perovskia abrotanoides* oils was evaluated in terms of percent inhibition of peroxidation in linoleic acid system [35]. Extract (5 mg) was mixed with linoleic acid solution (0.13 mL), ethanol (10 mL; 99.8%), sodium phosphate buffer (10 mL; 0.2 M; pH = 7) and diluted to 25 mL with distilled water. The solution was incubated at 40°C for 360 h and extent of oxidation was investigated using the colorimetric method [36]. Then at 0.2 mL sample solution, ethanol (10 mL; 75%), ammonium thiocyanate (0.2 mL; 30%) and 0.2 mL of ferrous chloride solution (20 mM in 3.5% HCl w/v) were added consecutively. After stirring for 3 min, the absorbance of mixture was calculated at 500 nm. A control was also performed only with linoleic acid (without any extract). The synthetic antioxidant such as BHT was used as positive control. Inhibition (%) of linoleic acid oxidation was investigated with the following equation:


#### Analysis of reducing power

The reducing power of the oils was determined according to the procedure described by Yen et al. [36] with little modification. The plant oils at various concentrations (2.5-10 mg) were mixed with sodium phosphate buffer (5 mL; 0.2 M) of pH 6.6 and potassium ferricyanide (5 mL; 1.0%). The mixture was heated for 20 min at 50°C. Then trichloroacetic acid (5 mL; 10%) was added and centrifuged at 980 rpm at 5°C for 10 min. Further, its upper layer (5 mL) was dissolved in 5 mL distilled water and finally freshly prepared ferric chloride (1 mL; 0.1%) was added. The absorbance was calculated at 700 nm and a result for each sample was recorded in triplicate.

### Antimicrobial assay

#### Microbial strains

*Bacillus cereus* ATCC 14579*, Escherichia coli* ATCC 25922*, Nitrospira sp.* locally isolated*, Staphylococcus epidermidis* ATCC 12229, *Staphylococcus aureus* API Staph tac 6736153 were used as bacterial strains and *Aspergillus niger* ATCC 10595*, Aspergillus flavus* ATCC *32612, Candida albicans* ATCC 10231 as fungal strains. The pure bacterial and fungal strains were obtained from the Department of Veterinary Microbiology, University of Agriculture, Faisalabad, Pakistan. The bacterial strains were cultured overnight at 37°C in nutrient agar (Oxoid, UK) while fungal strains were cultured overnight at 28°C using potato dextrose agar (Oxoid, UK).

#### Antimicrobial disc susceptibility test

Antimicrobial activity of the plant oils was determined by using the disc susceptibility test [37]. The discs (6 mm diameter) were impregnated plant oils (100 μL/disc) placed aseptically on the inoculated agar. Discs without injected samples served as a negative control, and Novidate (100 μL/disc) and Fungone (100 μL/disc) (both from Oxoid, UK) as positive control. The Petri dishes were incubated at 37 ± 0.1°C for 20–24 h and 28 ± 0.3 for 40–48 h for both bacteria and fungi. At the end of the period, the inhibition zones were measured. The positive antimicrobial activity was read based on growth inhibition zone.

#### Minimum inhibitory concentration (MIC)

The MIC of the plant oils was estimated following resazurin microtitre-plate assay reported by Sarker and contributors [38].

### Statistical analysis

All the aforementioned experiments were conducted in triplicate. Statistical comparisons were performed by one-way analysis of variance (ANOVA) followed by Dunnett’s t-test using SPSS version 12.0 (SPSSS Inc., Chicago, IL, USA). Probability values < 0.05 were considered to indicate significant difference.
